# Nasal Microbiota Profiling as a Predictive Secondary Tool for COVID-19 Diagnosis: The Critical Role of Taxonomic Resolution

**DOI:** 10.3390/microorganisms13030501

**Published:** 2025-02-24

**Authors:** Simon De Jaegher, Maria D’Aguanno, David Pinzauti, Manuele Biazzo

**Affiliations:** 1 The BioArte Ltd., Life Science Park, Triq San Gijan, 3000 San Gwann, Malta; simon.de.jaegher@hotmail.fr (S.D.J.); m.daguanno@thebioarte.com (M.D.); m.biazzo@thebioarte.com (M.B.); 2 Laboratory of Molecular Microbiology and Biotechnology (LAMMB), Department of Medical Biotechnologies, University of Siena, 53100 Siena, Italy

**Keywords:** SARS-CoV-2, human nasal microbiome, taxonomic resolution, Random Forest modelling, microbiome-based diagnostic

## Abstract

The SARS-CoV-2 pandemic has led to an urgent need for effective and rapid diagnostic tools. In the present study, we have evaluated the predictive diagnostic potential of nasal microbiota by analyzing microbial community structures at different taxonomic level resolutions—species, genus, family, order, class and phylum—using Random Forest modelling. A total of 179 nasal swabs from COVID-19-positive (n = 85) and COVID-19-negative (n = 94) individuals were sequenced using a full-length 16S rRNA sequencing (Oxford Nanopore) approach. During each iteration of the Random Forest model, the dataset was randomly split into a training set (70%) and a testing set (30%). Model performance improved with finer taxonomic resolution, achieving the highest accuracy at the Species level (AUROC = 0.821 ± 0.059) and a sensitivity of 55.6% at a specificity threshold of 90%. A progressive decline in AUROC and sensitivity was observed at broader taxonomic levels. Furthermore, Beta diversity analysis supported that microbial community structures are more distinct between COVID-19-positive and COVID-19-negative groups at finer taxonomic levels. These findings highlight the potential role of nasal microbiota profiling as a secondary diagnostic tool for COVID-19, particularly at species- and genus-level classification, and underscore the importance of high taxonomic resolution in microbiome-based diagnostics. However, limited by an uneven sample distribution and the lack of medical evaluations, further large-scale studies are needed before the nasal microbiota can be implemented in the clinical diagnostics of COVID-19.

## 1. Introduction

Coronavirus disease 2019 (COVID-19) is caused by Severe Acute Respiratory Syndrome Coronavirus 2 (SARS-CoV-2) and is commonly associated with symptoms such as fever, cough, shortness of breath (dyspnea) and fatigue [1]. The virus emerged in late 2019 and rapidly spread globally, resulting in a pandemic [2]. In response, significant research has focused on developing effective diagnostic and treatment strategies [3]. One promising diagnostic avenue is the analysis of the nasal microbiota, which may harbor microbial biomarkers associated with COVID-19 [4].

Most studies evaluating human nasal microbiota composition in the context of COVID-19 focus primarily on the species- [5] and genus-level [6] taxonomy classifications, or occasionally stopped at the family level [7]. Limitations in sequencing technology, poor sequencing quality, or inefficient analysis pipelines significantly impact taxonomy-level resolution, thereby restricting scientists’ ability to interpret the results. Hence, it is of extreme importance to determine at which taxonomic level the most accurate diagnostic information can be obtained. Armour et al. [8] evaluated the impact of taxonomic resolution for the prediction of colorectal cancer from microbiome data and suggested that increasing taxonomic resolution does not always enhance diagnostic accuracy, as data at finer levels can be too specific and lack overlap across individuals. Moreover, rather than always aiming for the highest resolution, researchers should prioritize finding the broadest taxonomic level that still provides a strong diagnostic signal [8].

We have recently evaluated species-level microbiome shifts in the distinguishing between COVID-19-positive and COVID-19-negative patients in Malta [9]. The results revealed significant differences in both Alpha and Beta diversity, with a trend toward increased abundances of opportunistic species in COVID-19 positive patients. Additionally, implementing a predictive generalized linear model (GLM) demonstrated that microbiome-based data can reliably differentiate between COVID-19-positive and -negative subjects, underscoring the potential of microbiome-driven data for distinguishing between healthy and disease states.

Building upon these findings, the present study employs more refined predictive models and specifically investigates the impact of taxonomic resolution on the ability to predict COVID-19 cases using nasal microbiome data. Based on Oxford Nanopore technology enabling full-length 16S rRNA sequencing and achieving accurate species-level classification [10,11], we evaluated the diagnostic accuracy across six taxonomic levels—species, genus, family, order, class and phylum—to determine which one provided the most effective resolution for diagnosing COVID-19 using nasal microbiome data. Moreover, following the methodology proposed by Timms et al. [12], microbial community structures associated with COVID-19 were assessed through Beta diversity differences across these taxonomic levels. This also aimed to identify the optimal taxonomic resolution for COVID-19 diagnostics based on nasal microbiome profiling.

## 2. Materials and Methods

### 2.1. Data Acquisition

Taxonomy data were gathered from a previous publication evaluating human nasal microbiota perturbations during the COVID-19 pandemic in Malta [9]. Raw fastQ sequencing reads were downloaded from the European Nucleotide Archive (ENA) repository, accession number PRJEB75547. Basecalled reads were analyzed as previously described [9] inferring taxonomic composition, and the resulting taxonomy tables were imported into RStudio (version 2024.12.0 Build 467, R version 4.3.3) [13]. The presence of potential contaminant species was assessed, filtering out samples with >3% relative abundance of *Faecalibacterium prausnitzii*, *Phocaeicola vulgatus*, *Agathobacter rectalis*, *Phocaeicola massiliensis*, *Ruminococcus bromii*, *Blautia wexlerae*, *Duodenibacillus intestinigallinarum*, *Phocaeicola coprocola* and *Parabacteroides merdae*. Potential contaminants were identified in negative controls and pruned utilizing the prune_taxa function from phyloseq (v. 1.46.0) [14]. Additionally, samples with fewer than 5000 mapped reads were discarded. Sample counts were normalized using the DESeq2 normalization function (DESeq2 v. 1.42.1) [15]. Potential confounding factors were previously assessed [9], revealing statistically significant differences related to ethnicity. To account for this, ethnicity was included as covariate in the analysis to ensure that the results were not biased by this factor.

### 2.2. Machine Learning

Random Forest (RF) predictive machine learning models were run using the R packages randomForest v. 4.7.1.1 [16] and pROC v. 1.18.4 [17]. The RF models were applied to the taxonomic data at six distinct taxonomic levels: species, genus, family, order, class and phylum. To ensure robustness and reproducibility, bootstrap resampling was enabled, selecting a fixed seed value of 100. During each iteration, the dataset was divided into a training set comprising 70% of the data and a testing set comprising 30% of the data. This 70:30 ratio allowed an optimal balance between training and testing needs, as it offered a robust training foundation that enhances feature learning and model reliability while still providing a reliable testing set size [18]. Receiver Operating Characteristic (ROC) curves were plotted for each taxonomic level based on the testing dataset. Model performance was then assessed by calculating the Area Under the ROC Curve (AUROC).

The reliability of the RF model was assessed evaluating sensitivity (true positive rate) and specificity (true negative rate) metrics [19]; probabilities from the testing dataset were collected for each iteration and subsequently used to calculate sensitivity at different specificity thresholds. The final sensitivity value—the RF model’s ability to detect true positives—was extracted when specificity was set at 90%, following the methodology outlined by Armour et al. [8]. To further validate statistical significance of RF model performance across different taxonomic levels, we conducted Analysis of Variance (ANOVA) tests using the aov function (stats v. 4.3.3) [20]. Tukey’s post hoc tests were also performed to identify specific differences between group averages, using the TukeyHSD function (stats v. 4.3.3) [20].

### 2.3. Beta-Diversity Analysis

Beta diversity analysis was conducted to validate the results achieved by the Random Forest model [12]. A Principal Coordinate Analysis (PCoA) ordination plot, based on the Bray–Curtis dissimilarity index, was used to evaluate Beta diversity at all identified taxonomic levels (R packages phyloseq v. 1.46.0 and ggplot2 v. 3.4.2) [14,21]. A Permutational Multivariate Analysis of Variance (PERMANOVA) test was performed to evaluate statistical significance using the adonis2 function (vegan v. 2.6.4) [22]. Additionally, permutation tests using the betadisper and permutest functions (vegan v. 2.6.4) [22] were also conducted to evaluate equal dispersion (intra group variability).

## 3. Results

A total of 179 patients (n = 179) were enrolled in the study, comprising 94 COVID-19-negative (52.51%) and 85 COVID-19-positive (47.49%) individuals [9]. After filtration, 65 COVID-19-negative and 70 COVID-19-positive samples were analyzed, classifying a total of 1268 bacterial species representing for 440 genera, 171 families, 81 orders, 29 classes and 17 phyla.

### 3.1. Machine Learning

**Figure 1:**
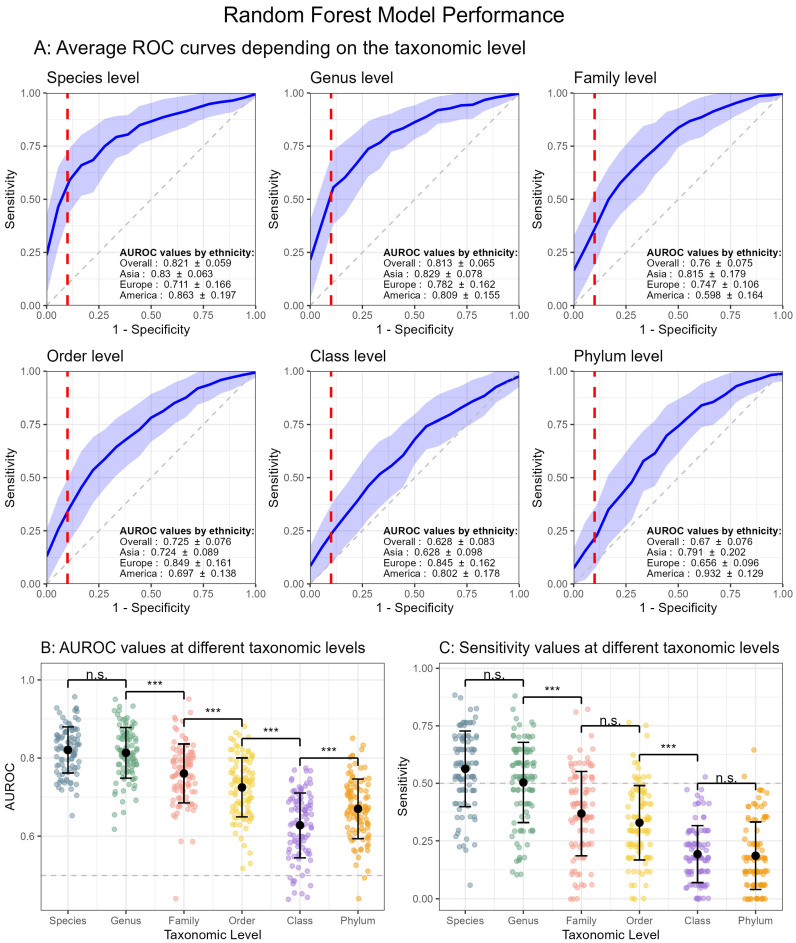
Random Forest model performance. Performance was evaluated across 100 runs using a RF model for different taxonomic levels—species, genus, family, order, class and phylum. (**A**) Average Receiver Operating Characteristic (ROC) curves for COVID-19 prediction. The blue line represents the mean ROC curve, and the shaded region indicates the standard deviation. The *x*-axis represents specificity, while the *y*-axis represents sensitivity. The red dashed line marks a specificity of 90%. Area Under the ROC Curve (AUROC) values are reported as mean ± standard deviation for the overall dataset and stratified by ethnicity (Asia, Europe and America). (**B**) AUROC values. (**C**) Sensitivity values measured at a specificity of 90%. Data are plotted as mean ± standard deviation, with taxonomic levels on the *x*-axis. The gray dashed line represents an AUROC or sensitivity of 0.5, which is equivalent to random classification [8]. Asterisks indicate significant differences between consecutive taxonomic levels (Tukey’s post hoc test), where *** *p*-value < 0.001 and n.s. = not significant.

The performance of the Random Forest model improved as taxonomic resolution increased, while maintaining discrimination (Figure 1). We first plotted Receiver Operating Characteristic (ROC) curves at each taxonomic level to illustrate the model’s ability to distinguish between COVID-19-positive and -negative cases (Figure 1A). The ROC curve flattens progressively from species level to phylum level, indicating a gradual reduction in discriminative power.

At the species level, the model achieved the highest AUROC (0.821 ± 0.059), while a trend toward lower AUROC values was observed as the taxonomic resolution decreased (Figure 1B). Specifically, the model’s performance at the genus (AUROC = 0.813 ± 0.065), family (AUROC = 0.760 ± 0.075), order (AUROC = 0.725 ± 0.076) and class (AUROC = 0.628 ± 0.083) levels showed a progressive decline compared to species-level classification. Notably, the AUROC measured at class level was lower than that measured at phylum level (0.670 ± 0.076), deviating from the overall trend. ANOVA and Tukey’s post hoc tests revealed significant differences between all taxonomic levels (*p* < 0.001), with the exception between species and genus (*p* = 0.98). These findings underscore the variability in model performance across different taxonomic resolutions, with the finest resolutions (species and genus) offering the best predictive performance. Incorporating ethnicity as a covariate in the RF model did not reveal substantial differences in AUROC values across different populations or taxonomic levels (Figure 1A). However, the uneven distribution of samples across ethnic groups requires cautious interpretation of these findings. Additionally, although the African cohort (n = 4) was included in the RF model, its insufficient sample size limited the ability to analyze it effectively, leading to its exclusion from the model.

Evaluating RF model sensitivity at different taxonomic ranks (Figure 1C), we estimated a sensitivity of 55.6 ± 20.5% at the species level, 50.7 ± 21.1% at the genus level, 40.9 ± 16.1% at the family level, 32.4 ± 16.1% at the order level, 22.7 ± 16.5% at the class level and 22.9 ± 15.5% at the phylum level. Importantly, only the species-level and genus-level models achieved a sensitivity greater than 50%. Sensitivity values measured for higher taxonomic levels did not exceed the 50% sensitivity threshold, suggesting that beyond the genus level, the model loses sensitivity (comparable to random classification [8]) and does not significantly improve the identification of COVID-19 individuals based on microbiome composition. ANOVA and Tukey’s post hoc tests revealed significant differences between all taxonomic levels (*p* < 0.001), except between species and genus (*p* = 0.09), family and order (*p* = 0.50) and class and phylum (*p* = 0.99).

### 3.2. Beta Diversity Analysis

**Figure 2:**
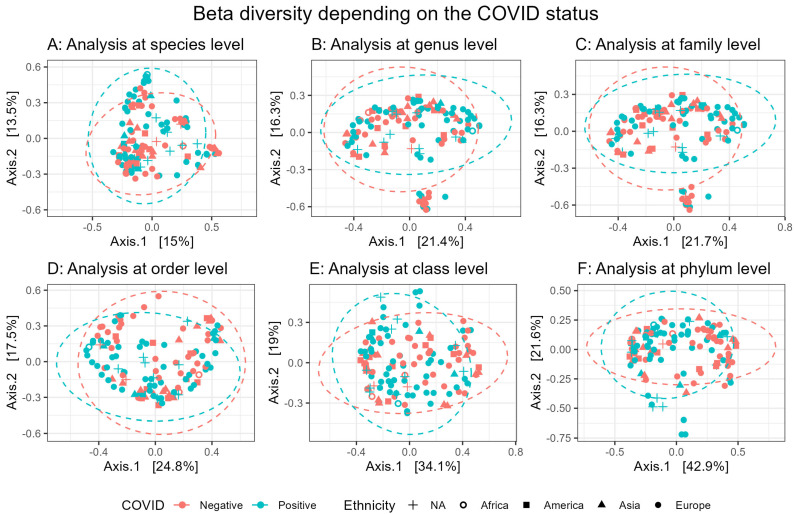
Beta diversity measured between COVID-19-positive (blue) and -negative (red) patients. Principal Coordinate Analysis (PCoA) plots show beta diversity (Bray–Curtis distance) computed at the species (**A**), genus (**B**), family (**C**), order (**D**), class (**E**) and phylum (**F**) taxonomic levels, with ellipses indicating 95% confidence intervals for each group. Samples were grouped according to their corresponding ethnicity, with those of unknown or missing ethnicity (NA) represented by a plus sign (+).

Beta diversity analysis examined how microbial community composition varied with COVID-19 status at different taxonomic levels. PCoA plots (Figure 2) were generated using the Bray–Curtis dissimilarity index. The proportion of variance explained by the axes suggested a reasonable representation.

PERMANOVA test (Table 1) yielded significant results at the species (*p* = 0.02), genus (*p* = 0.033) and family (*p* = 0.027) levels, indicating significant correlations between microbial community composition and COVID-19 status. Species-level resolution offered the greatest discriminatory power, highlighting fine-scale differences between groups. Genus and family levels also retained discriminatory power, but they provided less granularity compared to species-level resolution. The non-significant results of Permutest analysis (Table 1) indicate that the group differences observed using the PERMANOVA test were not influenced by unequal dispersion. Higher taxonomic levels, such as order, class and phylum, yielded insignificant PERMANOVA and Permutest results, thus suggesting that data at higher taxonomy resolutions are insufficient to distinguish between COVID-19-positive and -negative individuals. This trend reflects a loss of biological specificity as taxonomic resolution becomes coarser, emphasizing the importance of fine-scale analyses for detecting subtle community composition shifts related to COVID-19.

## 4. Discussion

The search for the optimal taxonomic resolution for detecting COVID-19 cases using nasal microbiota data is critical for developing a reliable tool to accurately distinguish positive from negative individuals. In this context, effective microbiome-based diagnostics depend on selecting the most informative level of taxonomic classification, as each level offers unique insights into microbial community structures. Our study demonstrates that species-level taxonomic resolution provides the highest diagnostic performance, as evidenced by Random Forest (RF) model accuracy and Beta diversity analyses.

In the present study, we applied a Random Forest classifier [8] to nasal microbiota data, evaluating sensitivity (true positive rate) and specificity (true negative rate) metrics across multiple taxonomic levels. Achieving an optimal balance between those two metrics is a fundamental challenge, as increasing sensitivity might lead to a decreasing specificity and vice versa [19]. The analysis demonstrated a notable increase in model performance as we move up the taxonomic resolution, with both species- and genus-level data achieving strong AUROC values (Figure 1A). Both levels significantly outperformed classifications at broader taxonomic levels (*p* < 0.0001), achieving an average sensitivity above 50% while maintaining a specificity of 90%, indicating that the RF model can effectively discriminate positive cases while also minimizing the rate of false positives. Moreover, further applying RF model at higher taxonomic level resolutions (family, order, class and phylum) showed sensitivity values below 50%. The result supports the hypothesis that at higher taxonomic levels, there are too few unique taxa and too much overlap to reliably differentiate positive and negative cases [8]. Despite including ethnicity as a covariate, we found no substantial impact on the RF model’s performance. To substantiate the previous findings, we evaluated Beta diversity analysis between COVID-19-positive and -negative patients. Significant group separation was observed at species, genus and family resolutions (Table 1), suggesting that microbial communities within these groups are more closely associated with COVID-19 status when examined at finer taxonomic resolutions. Computed Permutest values suggest that the results were not influenced by unequal dispersion (group variability). However, the observed high-degree overlaps in the 95% confident ellipses (Figure 2) suggested that, despite being significative, the magnitudes of differences in the microbiota composition are relatively small [9]. On the other hand, at the order, class and phylum levels, the observed differences in microbial community structure were not significant, reinforcing the idea that broader taxonomic levels fail to capture the subtle shifts induced by COVID-19. This could imply that COVID-19 affects specific microbial signatures in the nasal microbiota, signatures that become more evident when analyzed at finer taxonomic levels. Different species within the same genus can have unique associations with diseases, making it essential to identify biomarkers at the species level. Species-specific biomarkers provide more precise and practical tools for disease diagnosis compared to genus-level markers. These findings highlight that, in the context of COVID-19 diagnosis using nasal microbiome data, species-level taxonomic resolution could yield the most effective classifier.

This study has several limitations, including the lack of in-depth medical evaluation, which makes it impossible to fit our analysis, accounting for potential confounding factors such as prior or ongoing infections, medications or vaccination, which may result in microbiota composition shifts [23]. A substantial portion of study participants may have been vaccinated against COVID-19, though the exact number remains unknown. Further, individual variability in microbial community shifts—along with lifestyle, influences from environmental factors or pre-existing conditions—could also affect nasal microbiota composition in ways unrelated to COVID-19 [24,25]. Furthermore, as the data were previously de-identified and randomly selected [9], it was not possible to obtain a controlled and homogenous study population. This resulted in a diverse patient cohort in terms of ethnicity, which played a key role in microbiome variation (confounding factor). All these limitations highlight the need for future research with controlled vaccination data and adjustments for individual and environmental factors to strengthen the utility of microbiome data in diagnostic applications.

From a clinical perspective, nasal microbiota profiling holds potential as a supplementary diagnostic tool, complementing existing methods such as RT-qPCR. While RT-qPCR remains the gold standard due to its high sensitivity and specificity [26,27] microbiome-driven data could be effectively utilized as a secondary, confirmatory diagnostic test. By setting a high specificity threshold (90%), our model prioritizes reducing false positives, making it a valuable secondary diagnostic step to refine COVID-19 detection. In this role, it could effectively reduce false positives from a PCR test without compromising the need for rapid and accurate initial detection. This strategy allows the primary diagnostic to identify cases swiftly, while the secondary test (for ambiguous cases) could confirm positives more reliably, reducing unnecessary follow-ups and improving overall diagnostic accuracy. The feasibility of translating microbiota-based diagnostics into clinical practice will depend on advancements in sequencing technology. While 16S rRNA sequencing currently requires more time than conventional RT-qPCR, emerging nanopore-based rapid sequencing methods may enable point-of-care applications. Future research should focus on optimizing cost-effectiveness, turnaround time and automation to facilitate microbiota profiling as a real-time diagnostic approach.

Our findings align with broader trends in microbiome-based diagnostics across various fields. In a study conducted by Lin et al. [26], an RF machine learning model was employed for wastewater data to predict individual infections to COVID-19, showing the potential of machine learning algorithms in public health surveillance. Similarly, the same approach was also used in another study to explore the relationship between the oral microbiome and long COVID, identifying inflammatory bacterial taxa as potential biomarkers for persistent symptoms, thus highlighting the growing importance of microbiome-based diagnostics in understanding COVID-19 and its long-term effects [27]. Hence, the application of machine learning holds significant potential in harnessing nasal microbiota data for COVID-19 diagnosis. Zheng and colleagues [28] evaluated the role of gut microbiome biomarkers in the diagnosis of inflammatory bowel disease (IBD). Utilizing metagenomic data from a large cohort of fecal specimens, the authors identified species-specific bacterial biomarkers associated with IBD, achieving area under the curve (AUROC) values exceeding 0.90 across discovery and validation cohorts. This indicates a high level of accuracy in distinguishing IBD patients from healthy controls, outperforming traditional diagnostic methods like fecal calprotectin. Moreover, the characterization of bacterial biomarkers not only provided insights into microbial dysbiosis associated with IBD but also paved the way for more precise, personalized treatment options. The growing potential of microbiome-based diagnosis as a precise, non-invasive test has also been evaluated to predict cancer [29]. Introducing an artificial intelligence (AI) model leveraging Random Forest and transfer learning techniques to microbiome data, Xu et al. [29] demonstrated superior performance across over twenty cancer types using both blood and tissue samples (AUROC > 0.9 for most cancers). The incorporation of AI represents a significant leap toward personalized medicine: the ability to utilize microbiome data not only enriches the diagnostic landscape but also opens avenues for understanding the underlying biological mechanisms of cancer, emphasizing the potential of a non-invasive, multibacterial biomarker panel for early diagnosis and monitoring.

## 5. Conclusions

In light of these insights, the choice of taxonomic resolution in microbiota-based data for disease diagnosis should be made with careful consideration of the specific problem, data characteristics and analytical goals. To accurately distinguish COVID-19 cases based on nasal microbiota profiles, this study demonstrates that species-level taxonomic resolution is critical. Machine learning, exemplified by the Random Forest model, has proven effective in this pursuit. Indeed, by applying this model to full-length 16S rRNA sequencing data, we found that species- and genus-level data outperformed other taxonomic resolutions in diagnostic accuracy and sensitivity. Beta diversity analyses confirmed significant differences in microbial community structure at species, genus, family and order levels, underscoring their diagnostic potential. These results suggest that nasal microbiota profiling, especially at finer taxonomic resolutions, could serve as a valuable supplementary tool (confirmatory test) in COVID-19 diagnosis, complementing established methods like RT-qPCR.

This study provides preliminary results on the role of nasal microbiota in the diagnosis of COVID-19. To further validate the clinical applicability of nasal microbiome-based diagnostics, future studies should conduct larger, multi-cohort studies to assess reproducibility across different populations and explore machine learning integration with additional clinical data to improve predictive accuracy, investigating the impact of confounding factors such as vaccination status, patient symptoms, inflammatory markers, prior infections, chronic disorders and antibiotic usage on microbial signatures [23]. Moreover, rapid and accurate sequencing workflows that can enable real-time microbiome-based diagnostics in clinical settings should be developed. We believe that by addressing these challenges, nasal microbiota profiling could emerge as a valuable addition to the diagnostic landscape, not only for COVID-19 but also for other respiratory infections and broader applications in precision medicine.

While COVID-19 is the focus of this study, the broader implications of using microbiome data in diagnostics for other infectious diseases should be considered. The ability to identify specific microbial signatures associated with various pathogens could enhance disease detection in a wide range of infectious conditions, increasing the relevance and impact of microbiome-based diagnostics in clinical settings.

## Figures and Tables

**Table 1:** PERMANOVA and Permutest results assessing differences in community composition and dispersion across taxonomic levels. Asterisks refer to the computed *p*-value significance, where * *p*-value < 0.05.

Statistical Tests	Species	Genus	Family	Order	Class	Phylum
PERMANOVA (results and *p*-values)	F = 1.93	F = 2.02	F = 2.11	F = 1.99	F = 1.73	F = 2.33
	*p* = 0.02 *	*p* = 0.033 *	*p* = 0.027 *	*p* = 0.051	*p* = 0.122	*p* = 0.055
Permutest (results and *p*-values)	F = 0.19	F = 0.26	F = 0.37	F = 1.50	F = 0.96	F = 1.10
	*p* = 0.66	*p* = 0.61	*p* = 0.54	*p* = 0.22	*p* = 0.33	*p* = 0.30

## Data Availability

The data presented in the study are openly available in the European Nucleotide Archive (ENA) repository, PRJEB75547.

## Abbreviations

The following abbreviations are used in this manuscript:

SARS-CoV-2	Severe Acute Respiratory Syndrome Coronavirus 2
COVID-19	Coronavirus disease 2019
RF	Random Forest
ROC	Receiver Operating Characteristic
AUROC	Area Under the ROC Curve
